# Discontinuation of Passive Immunization Is Safe after Liver Transplantation for Combined HBV/HDV Infection

**DOI:** 10.3390/v13050904

**Published:** 2021-05-13

**Authors:** Ramin Raul Ossami Saidy, Irina Sud, Franziska Eurich, Mustafa Aydin, Maximilian Paul Postel, Eva Maria Dobrindt, Johann Pratschke, Dennis Eurich

**Affiliations:** Department of Surgery, Campus Virchow-Klinikum and Campus Charité Mitte, Charité—Universitätsmedizin Berlin, Augustenburger Platz 1, 13353 Berlin, Germany; raminraul@web.de (R.R.O.S.); irina.sud@charite.de (I.S.); franziska.eurich@charite.de (F.E.); mustafa.aydin@charite.de (M.A.); maximilian.postel@charite.de (M.P.P.); eva-maria.dobrindt@charite.de (E.M.D.); johann.pratschke@charite.de (J.P.)

**Keywords:** liver transplantation, HBV, HDV, passive immunization, hepatitis

## Abstract

Patients after LT due to combined HBV/HDV infection are considered to be high-risk patients for recurrence of hepatitis B and D. To date, life-long prophylaxis with hepatitis B immunoglobulin (HBIG) and replication control with nucleos(t)ide analogs (NA) remains standard. We examined the course of 36 patients that underwent liver transplantation from 1989 to 2020 for combined HBV/HDV-associated end-stage liver disease in this retrospective study. Seventeen patients eventually discontinued HBIG therapy for various reasons. Their graft function, histopathological findings from routine liver biopsies and overall survival were compared with those that received an unaltered NA-based standard regimen combined with HBIG. The median follow-up was 204 and 227 months, respectively. The recurrence of HBV was 25% and did not differ between the groups of standard reinfection prophylaxis NA/HBIG (21.1%) and HBIG discontinuation (29.4%); (*p* = 0.56). No significant differences were found regarding the clinical course or histopathological aspects of liver tissue damage (inflammation, fibrosis, steatosis) between these two groups. Overall, and adjusted survival did not differ between the groups. Discontinuation of HBIG in stable patients after LT for combined HBV/HDV did not lead to impaired overall survival or higher recurrence rate of HBV/HDV infection in this long-term follow-up. Therefore, the recommendation of the duration of HBG administration must be questioned. The earliest time of discontinuation remains unclear.

## 1. Introduction

The prevalence of acute and chronic infection with hepatitis B virus (HBV) remains high worldwide [1]. Modern therapy strategies focus on replication control with nucleos(t)ide analogs (NA) or interferons (IFN) in patients with chronic HBV infection to prevent end-stage liver disease (ESLD) in terms of cirrhosis and hepatocellular carcinoma (HCC) [2]. Aggravation of infections can be caused by co- or superinfection of hepatitis D virus (HDV) with rapid progression of hepatitis to end-stage liver disease (ESLD) [3]. In patients with acute or chronic HBV/HDV infection and resulting liver failure, liver transplantation (LT) may be necessary, and after transplantation, reinfection prophylaxis is needed [4,5].

While the combination of NAs and passive immunization with hepatitis B immunoglobulins (HBIG) revolutionized the effective control of reinfection and replication, recent studies indicated NA monotherapy after LT for HBV-associated ESLD might be efficient with comparable outcomes in certain subgroups [6]. As HBIG therapy was initially recommended in a life-long manner, the evaluation of the reduction and eventual discontinuation entails significant costs and lack of patient’s adherence, as the substance needs to be administered intravenously or subcutaneously [7,8,9,10].

Due to the aggressive course of uncontrolled HBV/HDV reinfection, however, guidelines classify patients with combined HBV/HDV infection as “high-risk” and recommend lifelong combination prophylaxis (NA and HBIG) [5,11]. However, some state that discontinuation can be evaluated after 1 year after LT [11]. Cholongitas et al. analyzed 34 patients with HBIG discontinuation, reporting only 2 patients with reactivation of HBV/HDV infection with a median follow-up time of 30 months after discontinuation. Here, early discontinuation was identified as a risk factor for recurrence of combined HBV/HDV. However, the long-term relevance and impact of HBV/HDV infection after LT is unknown.

Data for this special collection are scarce. Thus, concrete circumstances and feasibility of HBIG withdrawal and course after LT in patients with combined HBV/HDV infection remain unclear, although a prolonged combination regimen of 12 months after LT before discontinuation seems favorable [12]. Therefore, we performed a retrospective analysis of all patients who had undergone LT for HBV/HDV-associated ESLD regarding their reinfection prophylaxis regimen to understand the role of HBIG administered long-term compared to HBIG discontinuation.

## 2. Patients and Methods

Between 1988 and 2020, 3177 LTs were performed to treat various liver diseases at our transplant center. Among these, 36 patients (1.3%) were identified with combined HBV/HDV-associated ESLD leading to LT. Follow-up was conducted via our outpatient department for transplantation with regular clinical examinations and laboratory tests at intervals ranging from twice a week to once in 12 weeks in a time-dependent manner after LT.

Further, routine biopsies per protocol were performed at 1, 3, 5, 7, 10, and so on years for an indefinite duration and on suspected rejection, hepatitis or unclear elevation of aminotransferases (TA)/dysfunction of transplant as part of the routine aftercare.

Levels of (TAs) from laboratory tests were dichotomized into normal and elevated according to threshold parameters; elevation of either one of TA or both was considered as pathological.

Histopathological assessment of inflammation grade and fibrosis stage was performed using the classification proposed by Desmet and Scheuer [13]. Thus, grades of inflammation were classified as 0—no inflammation; 1—minimal; 2—mild; 3—moderate; and 4—severe. Fibrosis was staged as follows: 0—absent; 1—mild portal fibrosis; 2—moderate with few incomplete portal septa; 3—numerous portal septa without architectural disturbances and 4—cirrhosis. Steatosis was assessed as follows: 1—<30%, 2—<60% and 3—>60%.

Reinfection and monitoring of HBV/HDV were conducted with polymerase chain reaction (PCR) from blood samples and established serological markers. The diagnosis of reinfection was defined as the reappearance of HBs antigen (HBsAg) or HBV-DNA, or HDV-RNA, respectively.

The standard approach to hepatitis B and D reinfection prophylaxis included lifelong administration of antivirals. Until the 1990s, immunoglobulins with a target titer of more than 500 U/L were administered intravenously. Later, NAs with a lower barrier to resistance, such as lamivudine was administered first, followed by more potent substances, such as tenofovir and entecavir as a basis in addition to HBIG (Biotest, Dreieich, Germany) with a target level of at least 100 U/L.

For comparison, two groups were formed retrospectively based on a regimen of HBV/HDV-reinfection prophylaxis after LT. Patients who underwent a standard regimen with a life-long combination of HBIG and NA were assigned to a standard reinfection prophylaxis group (“standard” group). Patients eventually discontinuing HBIG therapy for various reasons were assigned to a “discontinuation” group. The oral application of NAs was continued.

Descriptive analysis was used to calculate the median. For continuous variables, *t*-test or—in the case of skewed data—Mann–Whitney *U*-test were performed. Wilcoxon signed-rank test was applied to detect differences in paired categorical variables, and for comparison of categorical variables, cross tables were used. Kaplan–Meier analysis with Log-rank-test was performed to compare and illustrate survival differences and mean with 95% confidence interval (CI) were given. A *p*-value (two-sided) of <0.05 was considered to be statistically significant. SPSS software version 26 (IBM, Armonk, NY, USA) was used for all statistical analyses.

The study was performed retrospectively according to the Professional Code of the German Medical Association (article B.III.§15) based on the World Medical Association’s Declaration of Helsinki and was approved by the Ethics Committee of Charité Universitätsmedizin Berlin (protocol code EA1/035/21).

## 3. Results

Among 3177 liver transplantations in 2840 patients at our transplant center, 36 (1.3%) patients were identified who had undergone LT for HBV/HDV-associated ESLD and were closely followed up at our center for more than 30 years highlighting the rarity of this special constellation.

Patients were retrospectively divided into two groups based on course: the “discontinuation” group eventually discontinuing HBIG therapy and the “standard” group receiving the standard regimen with life-long combination therapy.

Discontinuation of HBIG was documented in *n* = 17 (47.2%) patients due to various reasons (patients’ incompliance/wish/non-adherence) with median time after LT of 72 (0–312.0) months. Median follow-up after discontinuation was 204 (7.0–360.0) months.

In *n* = 17 (89.5%; standard) and *n* = 15 (88.2%; discontinuation), respectively, HBsAg or HBV-DNA were positive at the time of LT, indicating an active HBV infection. Titers of HBV-DNA were available in three patients in the standard reinfection prophylaxis group with a median of 60,111.0 (12.0–220,000.0) IE/mL and in six patients in the discontinuation group with a median of 30,250.0 (10–500,000) IE/mL. Before LT, only four patients in each group had undergone specific therapy for HBV/HDV infection with NA. From these, one patient suffered from a recurrence of HBV. Analysis of the impact of antiviral therapy pre-LT on recurrence of HBV infection did not show significance (*p* = 0.36).

HBIG levels were sufficient with titers >100 U/L in both groups, and the level of Anti-HBs decreased beyond the level of detection in *n* = 13 (76.5%) but remained elevated in four (23.5%) patients after discontinuation. Hepatitis C virus (HCV) coinfection was present in seven patients in the standard reinfection prophylaxis group and in four patients in the HBIG discontinuation group and did not differ with statistical significance (*p* = 0.43).

All patients received combination therapy to control HBV/HDV reinfection with HBIG and nucleos(t)ide analogs except for *n* = 5 patients in the discontinuation group where HBIG monoprophylaxis was conducted.

Recent immunosuppressive (IS) regimen predominantly consisted of calcineurin inhibitors (CNI) with the combination of mycophenolate mofetil (MMF). Other combinations (e.g., prednisolone, mTOR-Inhibitors) were rare.

Seven patients (36.8%; standard group) and three patients (17.6%; discontinuation group) were deceased at the moment of this analysis. The most frequent causes of death were cardiovascular events/thromboembolism (*n* = 5) and neoplasia (*n* = 4). Only one patient in the standard regimen group standard died of graft dysfunction eight years after LT associated with chronic rejection. Here, reactivation of virus hepatitis occurred 15 months after LT despite combination therapy, and adherence to therapy was excellent. There were no statistically significant differences in general patients’ characteristics between the two groups (Table 1).

### 3.1. Recurrence of HBV/HDV

After transplantation, reactivation of HBV infection was diagnosed with new-onset of HBV-DNA in PCR and was found in four (21.1%) patients (standard group) and in five patients (29.4%) (discontinuation group), respectively. Only one (5.9%) patient in the discontinuation group suffered from combined HBV/HDV reinfection one year after LT. Seven reinfections (19.4%) occurred while combination therapy was administered. In two patients (11.8%), reinfection with HBV occurred after discontinuation of HBIG, and in both, HBIG therapy did not exceed 6 months after LT (3.6 and 6 months, respectively).

In all patients with recurrence, HBIG was discontinued and therapy was switched to high-genetic-barrier NA.

**Figure 1:**
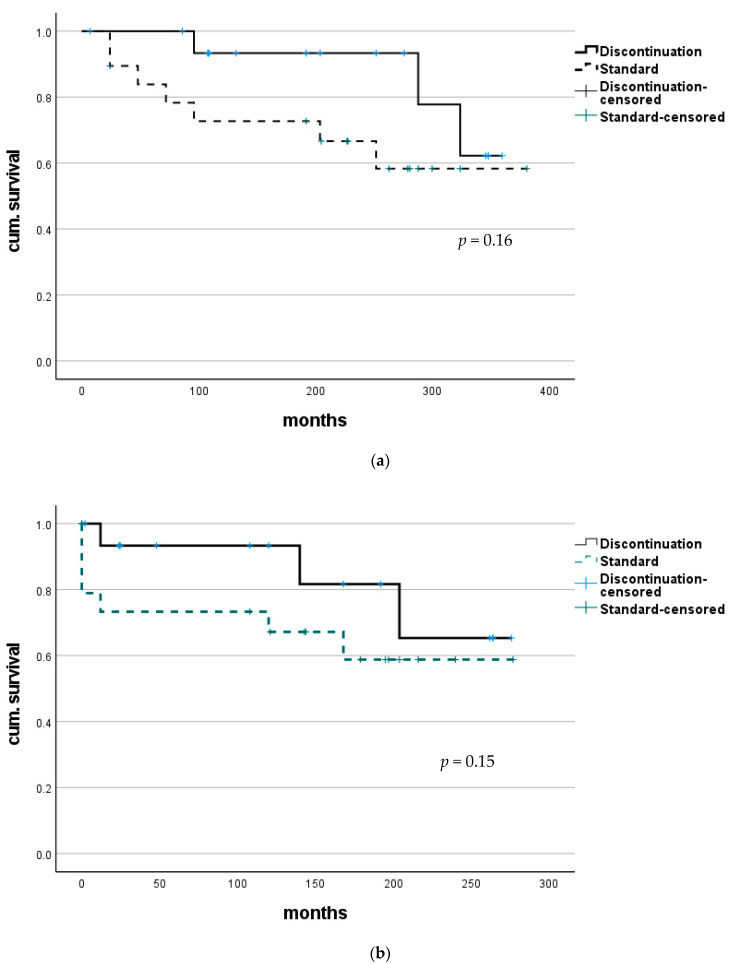
Survival after LT for combined HBV/HDV infection. (**a**) No statistically significant difference in overall survival was found between patients with continuous HBIG therapy (group Standard) and those where HBIG was eventually discontinued (group discontinuation). (**b**) Comparing survival after adjusting to a median time of discontinuation (72 months) to better evaluate course after HBIG discontinuation also did not show any significant difference.

In seven patients, HBV-DNA was undetectable under this regimen within months. In the discontinuation group, one patient (5.9%) suffered from combined HBV/HDV infection and occasionally reappearing HBV-DNA/HDV-RNA was found for up to 14 years after LT. Here patients’ compliance was impaired, and medication adherence was reduced. Finally, follow-up 22 years after LT. However, PCR for HBV/HDV was negative. Another patient from the discontinuation group had to undergo re-transplantation due to HBV infection 18 months after the initial LT (Figure 1). Mann–Whitney-U test showed no significant impact of duration of HBIG therapy on reactivation with median administration of HBIG in patients with recurrence of 117.6 (3.6–228.0) months and 84.0 (0–381.0) months in those without (*p* = 0.52).

### 3.2. Histopathological Findings

**Figure 2:**
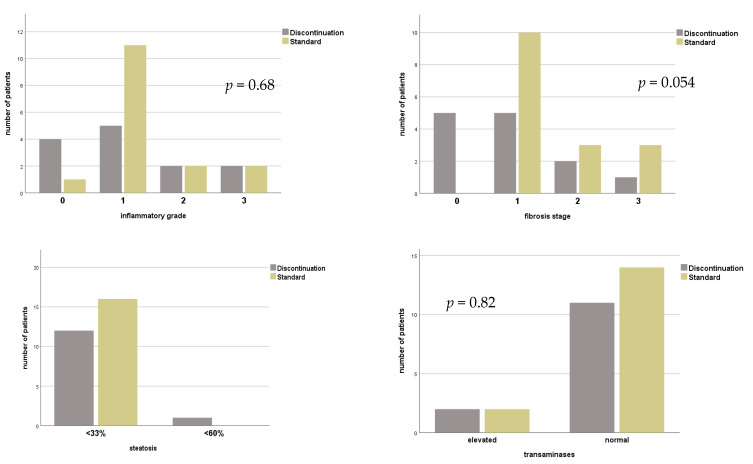
Histopathological findings and level of transaminases of patients with or without continuous HBIG prophylaxis for combined HBV/HDV infection before liver transplantation. Group standard—life-long combination of HBIG with NA; group discontinuation—discontinuation of HBIG.

Histopathological findings from routine biopsies were available in 16 (84.2%) patients with continuous HBIG therapy. (Figure 2) Median time from LT was 132.5 (12–204) months, and complete absence of inflammation was seen in one patient (5.3%), minimal inflammation in 11 (57.9%) and mild or moderate inflammation in two (10.5%) each. Only mild portal fibrosis was found in 10 (52.3%), while three patients (15.8%) were reported with grade 2 and another three (15.8%) with grade 3. Liver steatosis was below 30% in all samples. Concurrent blood levels of aminotransferases were normal in 14 (73.7%) patients.

In patients where HBIG therapy was eventually discontinued, *n* = 13 (76.5%) biopsy results under combination therapy were documented with median time after LT of 60 (1–240) months. In three patients (17.6%), inflammation was absent; in eight patients (47.1%) only minimal inflammation was confirmed. Grade 2 inflammation was seen in two patients (11.8%). No fibrosis was seen in *n* = 4 (23.5%), stage 1 in *n* = 6 (35.3%) and stage 2 in three patients (17.6%). Most *n* = 12 (70.6%) were classified with steatosis below 30%. Only one (5.3%) with up to 60%. Aminotransferases were elevated in four (21.5%) patients.

**Figure 3:**
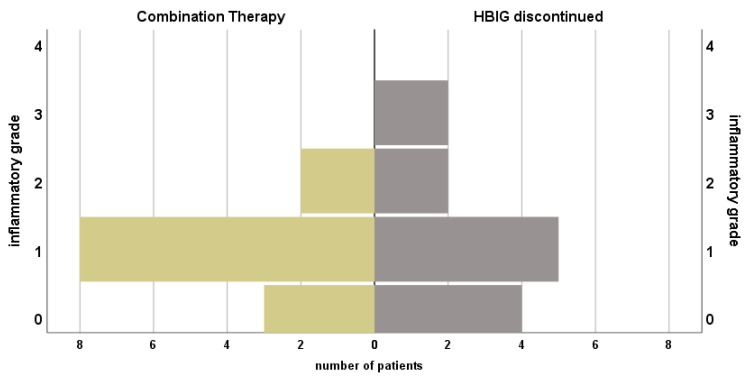

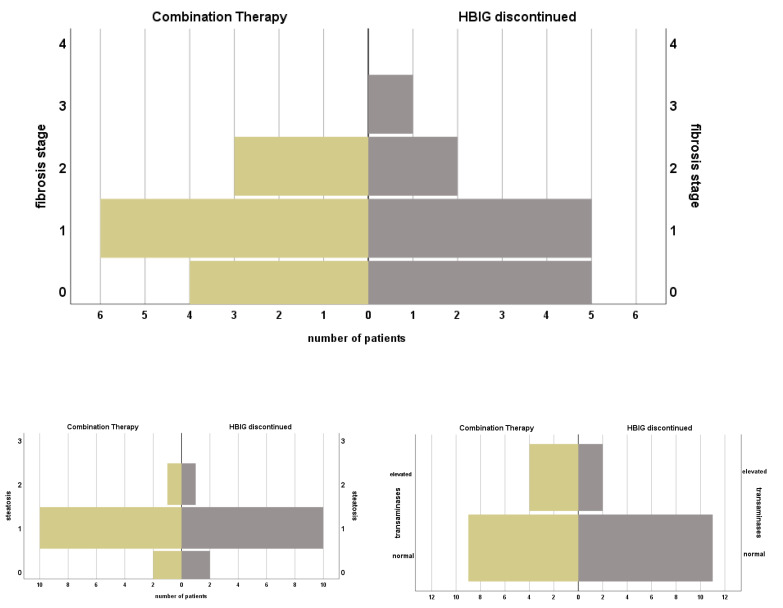
Course of histopathological findings and serum transaminases of patients under prophylactic combination therapy with NA + HBIG and after discontinuation of HBIG. Median time between biopsies: 120.0 (1.0–360.0) months.

Histopathological findings of biopsies after discontinuation of HBIG were available in *n* = 13 (76.5%) and median time after change of regimen was 120.0 (1–360.0) months. In four (23.5%) patients no inflammation and in five (29.4%) inflammation grade 1 was found. Mild and moderate inflammation were described in 2 (11.8%) patients, respectively. No fibrosis was reported in *n* = 5 (29.4%), stage 1 in 5 patients (29.4%), moderate in two specimen (11.8%) and one patient (5.9%) were found with stage 3 fibrosis. Liver steatosis was below 30% in *n* = 12 (70.1%). Elevated TAs were found in two (11.8%) patients. Wilcoxon test showed no significant change in histopathological grading of inflammation (*p* = 1.0), fibrosis (*p* = 0.53), steatosis (*p* = 0.56) or elevation of TAs (*p* = 0.56) when comparing status before and after HBIG discontinuation, see Figure 3.

Similarly, findings between groups did not differ during combination therapy (grade of inflammation: *p* = 0.29; stage of fibrosis; *p* = 0.051; extent of liver steatosis; *p* = 0.13 pathological TAs; *p* = 0.23) and time of biopsy after LT was comparable (discontinuation 81.2 ± 63.0 months vs. standard 121.6 ± 63.0 months; *p* = 0.09).

No statistically significant difference was found for these parameters when comparing biopsies of standard with those after discontinuation of HBIG (grade of inflammation: *p* = 0.68; stage of fibrosis; *p* = 0.054; extent of liver steatosis; *p* = 0.13; pathological TAs; *p* = 0.82), see Figure 2.

### 3.3. Survival

Survival analysis showed no significant difference in overall outcome between these groups (median survival of the discontinuation group 270.1 (CI 203.3–337.0) months vs. standard group 325.6 (CI 288.4–362.8) months; *p* = 0.15). To compare courses after the splitting of groups—discontinuation of HBIG—survival analysis was adjusted using median time of discontinuation (72.0 months) to define a new starting point after LT. In this analysis, survival also did not differ with statistical significance (median survival the discontinuation group 132.0 (CI 216.0–290.0) months vs. standard group 155.0 (144.8–267.6) months; *p* = 0.16); see also Figure 1.

For a paired analysis of outcome after HBIG discontinuation, *n* = 7 patients from the standard group were matched with *n* = 7 patients from the discontinuation group (see Table 2). Thus, the median time after discontinuation was 80.0 (0–312.0) months after LT. Survival analysis was again compared overall and adapted to the time of discontinuation. Similar, survival for patients in both groups did not differ overall (*p* = 0.09), but when adjusted to the time of discontinuation of combination therapy (80.0 months), a significant difference in survival was seen in the discontinuation group with a median of 196.0 (30–280) and 144.0 (0–172) months in the standard prophylaxis group (*p* = 0.04).

## 4. Discussion

This is a retrospective analysis of HBV/HDV reinfection prophylaxis mode in liver transplant patients from a large volume transplant center with a long observation period. In analogy to previously published studies from our department, among others, the long observation period of the patient courses was used to determine whether the administration of HBIG should actually be performed lifelong in the so-called risk groups for reactivation of hepatitis B and D [14]. The long observation period and the differences in patient compliance were used to identify and derive indications for further possibilities of reinfection prophylaxis in the future. The findings support the notion that, contrary to current recommendations, HBIG application is probably not intended to be lifelong and may not even be necessary at this level of breadth because the antiviral effect has shifted to NAs over time.

Discontinuation of HBIG while continuing NA as the basis of HBV/HDV reinfection prophylaxis has several advantages for both the patient, staff, and healthcare system, especially during periods of restrictive patient contact.

Lack of disadvantage at the level of laboratory chemistry, histology and survival as outlined in our study is associated with relevant cost-savings. On average, per patient and per year, about 4 routine physician visits, 17,000–26,000 units of HBIG (corresponding to about 17,000–26,000 Euros) are saved.

Certain limitations have to be discussed: Given the rare occurrence of the disease, our retrospective study describes a heterogeneous collective of 36 patients over 30 years. Only 17 eventually discontinued HBIG therapy, reflecting the current recommendation of the guideline for HBV infection in Germany from 2011 towards a life-long regimen [15]. In addition, this study is not able to further specify the period for HBIG therapy after LT. To our knowledge, only Cholongitas et al. examined a collective similar to this in our study to reevaluate the role of HBIG. From their collective, they concluded, HBIG therapy should be continued for at least 12 months, as there was a significantly shorter span of administration in patients with reinfections (median 9 vs. 28 months). We found reactivation in two patients, where HBIG therapy was discontinued within 6 months after LT, further corroborating the notion that a certain duration of this regimen should be adhered to. Here, a correlation between the extent of immunosuppression—that often is highest in the early post-transplant setting—might exist [16]. While combined HBV/HDV reinfection after LT used to be a feared complication with the imminent threat of graft loss or fatal outcome, it is now very well manageable, and patients‘ course is comparable to HBV reinfection, reflecting the progress that antiviral therapies have undergone [17,18,19,20,21]. We only encountered one combined HBV/HDV reinfection in a patient with difficult compliance. Still, treatment was conducted successfully, and 22 years after LT, infection was not detectable anymore.

We found a reactivation of HBV infection after LT of up to 25% of patients in our collective, as previously described for our overall collective of patients after LT for HBV infection, while other studies described a lower incidence for combination therapy after LT [6,22]. Recently, we published that HBV reinfection per se, if controlled with NA, does not lead to poorer patient survival when follow-up is appropriate [22].

In concordance with Cholongitas et al., we did not find a statistically significant impact of discontinuation of HBIG therapy in stable liver transplant recipients in the time of highly effective NA-based therapy regimens. While the median period from LT to discontinuation was longer in our study (72 months), so was median follow-up with histological confirmation of no progression to cirrhosis or impact on patients’ survival.

A trend towards improved survival for patients with discontinuation of HBIG was observed, and after paired matching and adjustment of survival for the time of discontinuation, the difference reached statistical significance. However, this is based on a selection bias. As in our study, most patients discontinued HBIG after long-term administration at different time points after LT. Thus, it is likely, that a better outcome may be overestimated, but not-inferiority can be assumed safely. Safety of HBIG discontinuation for patients with only HBV infection has been described, and guidelines have acknowledged the possibility for these “low-risk” patients [11,14,23,24].

A recent study described the post-transplant course of 104 patients with HBV/HDV infection before LT. Here, reinfection occurred in 13.4% despite the combination of NA and HBIG, but the infection did not affect overall survival [18]. The authors concluded that modern NA therapy could control reactivation with a favorable outcome. Moreover, in this study, additional evidence was collected that, based on strong NA-backbone therapy, regimen towards reinfection prophylaxis can and should safely limit HBIG therapy in the post-transplant context. Still, HBIG remains an important tool for reinfection prophylaxis in the early post-transplant setting and in its different indications in patients with HBV infection, such as post-exposure prophylaxis or in patients without sufficient boost by vaccination, but with ongoing risk of infection are undisputed [5].

The ideal time of discontinuation in the cohort of HBV/HDV reinfection prophylaxis after LT remains uncertain; thus, the focus on future studies regarding this topic should be “how” rather than “if”. The classification of patients into high and low-risk groups loses its meaning since reinfection, if detected in time, is neither associated with worse histology nor with limited survival, as shown in this analysis.

## 5. Conclusions

Discontinuing HBIG while continuing NA as the basis of HBV/HDV reinfection prophylaxis could be safe, cost-effective, contact-reducing, and without patient harm. However, this should be verified in prospectively organized randomized studies.

## Figures and Tables

**Table 1:** Patient characteristics. CNI—calcineurin inhibitor; LT—liver transplantation; HCC—hepatocellular cancer; mTOR—mTOR Inhibitor; MMF—mycophenolate mofetil; HBIG—hepatitis B immunoglobulin; NA—nucleos(t)ide analog; standard group–life-long combination of HBIG with NA; discontinuation group—NA-based and discontinuation of HBIG.

Patient Characteristic	Standard (NA + HBIG)	Discontinuation (NA + HBIG-)	*p*
Total patients (%)			0.74
Male	9 (47.4)	9 (52.9)	
Female	10 (52.6)	8 (47.1)	
Median age at LT (min–max)	45 (32–59)	42 (20–62)	0.95
HBV/HDV infection (%)	19 (100)	17 (100)	
HBsAG+/HBV-DNA+ at LT	17 (89.5)	15 (88.2)	0.91
HBV reinfection after LT	4 (21.1)	5 (29.4)	0.56
HDV reinfection after LT	0 (0)	1 (5.9)	0.11
HCV infection at LT (%)	7 (36.8)	4 (23.5)	0.43
HCV reinfection after LT	3 (42.9)	2 (50.0)	
HCC at LT (%)	4 (21.1)	7 (41.2)	0.43
Re-transplantation (%)	2 (10.5)	2 (11.8)	0.26
Rejection	0 (0)	1 (5.9)	
Reinfection	0 (0)	1 (5.9)	
Surgical complications	2 (10.5)	0 (0)	
HBV/HDV prophylaxis after LT (%)			0.11
NA: low genetic barrier	10 (52.6)	9 (52.9)	
NA: high genetic barrier	7 (36.8)	4 (23.5)	
IFN	2 (10.5)	1 (5.9)	
HBIg monotherapy	0 (0)	5 (29.4)	
Median HBIG titer (U/L) during combination therapy in (min–max)	215.5 (140–845)	199 (102–1000)	0.40
Recent immune suppression (%)			
CNI	17 (68.4)	15 (58.8)	0.55
MMF	8 (42.1)	7 (41.2)	0.92
mTOR	0 (0)	1 (5.9)	0.28
others	1 (5.3)	1 (5.9)	0.93
combination			
CNI/MMF	7 (36.8)	5 (29.4)	
others	1 (5.3)	1 (5.9)	
Median follow-up after LT (min–max)	227 (24–381)	204 (7–360)	0.69
Median time of HBIG discontinuation after LT (min–max)	n.a.	72 (0–312)	n.a.
Median time after HBIG discontinuation (min–max)	n.a.	120 (6–360)	n.a.
Deceased (%)	7 (36.8)	3 (17.6)	0.2

**Table 2:** Matched pairs used for analysis. Standard—life-long combination of HBIG with NA; discontinuation—discontinuation of HBIG; LT—liver transplantation; F—female; M—male.

Pair	Standard (NA + HBIG)	Matched Parameters	Discontinuation (NA + HBIG-)
	M	Sex	M
1	39	Age at LT	38
	1989	Year of LT	1991
	M	Sex	M
2	48	Age at LT	51
	2003	Year of LT	2004
	F	Sex	F
3	34	Age at LT	32
	1990	Year of LT	1992
	M	Sex	M
4	47	Age at LT	47
	2003	Year of LT	2002
	M	Sex	M
5	37	Age at LT	32
	1990	Year of LT	1989
	F	Sex	F
6	34	Age at LT	34
	1997	Year of LT	2000
	F	Sex	F
7	42	Age at LT	44
	2010	Year of LT	2012

## Data Availability

The data presented in this study are available on request from the corresponding author. The data are not publicly available due to stipulation of the local ethics committee and local data policy.

## Abbreviations

CI	Confidence interval
CNI	Calcineurin-inhibitor
ESLD	End-stage liver disease
HBIG	Hepatitis B immunoglobulin
HBsAg	Hepatitis B surface antigen
HBV	Hepatitis B virus
HCC	Hepatocellular carcinoma
HDV	Hepatitis D virus
IFN	Interferon
IS	Immunosuppression
LT	Liver transplantation
MMF	Mycophenolate mofetil
NA	Nucleos(t)ide analog
PCR	Polymerase chain reaction
TA	Aminotransferase/transaminase
